# Machine Learning for Exposure-Response Analysis: Methodological Considerations and Confirmation of Their Importance via Computational Experimentations

**DOI:** 10.3390/pharmaceutics15051381

**Published:** 2023-04-30

**Authors:** Rashed Harun, Eric Yang, Nastya Kassir, Wenhui Zhang, James Lu

**Affiliations:** 1 Genentech Inc., South San Francisco, CA 94080, USA; 2 Department of Biomedical Informatics, Harvard Medical School, Boston, MA 02115, USA

**Keywords:** machine learning, exposure-response, causal inference

## Abstract

Exposure-response (E-R) is a key aspect of pharmacometrics analysis that supports drug dose selection. Currently, there is a lack of understanding of the technical considerations necessary for drawing unbiased estimates from data. Due to recent advances in machine learning (ML) explainability methods, ML has garnered significant interest for causal inference. To this end, we used simulated datasets with known E-R “ground truth” to generate a set of good practices for the development of ML models required to avoid introducing biases when performing causal inference. These practices include the use of causal diagrams to enable the careful consideration of model variables by which to obtain desired E-R relationship insights, keeping a strict separation of data for model-training and for inference generation to avoid biases, hyperparameter tuning to improve the reliability of models, and estimating proper confidence intervals around inferences using a bootstrap sampling with replacement strategy. We computationally confirm the benefits of the proposed ML workflow by using a simulated dataset with nonlinear and non-monotonic exposure–response relationships.

## 1. Introduction

Exposure-response (E-R) analysis is an integral part of clinical drug development and can be highly informative for dose-selection [1]. Accounting for confounders, factors that simultaneously affect both exposure and response, is key to E-R analysis. While the issue of confounded E-R relationships is well established for monoclonal antibodies (mAbs) in the treatment of inflammatory indications such as rheumatoid arthritis and inflammatory bowel disease (IBD) [2], there are a set of good practices that have been proposed to ensure the validity of conclusions that are drawn from such E-R analyses [3].

While many methodologies exist for performing E-R analyses [1], the advent of machine learning (ML) and deep learning (DL) [4,5,6] opens up a novel approach. To address the presence of confounders that may affect either exposure and/or response in nonlinear manners and to explore the potential benefits of DL in E-R analysis, the FDA authors [7] generated synthetic data that involved complex, nonlinear relationships and compared traditional logistic regression with DL models. Using synthetic data, the DL approach more accurately adjusted for confounders such that the identified E-R was in good agreement with the known ground truth [7]. Additionally, when estimating heterogeneous treatment effects, the potential benefits of ML in handling covariates that manifest nonlinear relationships in the presence of data noise was also demonstrated [8].

More recently, a number of different statistical and ML models were applied to analyze the exposure-response (E-R) for efficacy from oncology clinical trials [9]. Specifically, a tree-based ensemble ML algorithm (XGBoost) was compared against logistic regression and the Cox proportional hazards model with elastic net penalty, for binary and survival clinical outcomes, respectively. The methodology is based upon the computation of Shapley Additive Explanations (SHAP) [10], which has been used to quantify the contribution of explanatory variables to hazard ratios in the setting of ML models of survival data [11]. While SHAP analysis has become widely applied in a number of different pharmacometrics modeling applications (e.g., [12,13]), care is needed in the ML workflow to avoid drawing biased estimates of the E-R relationship and to ensure that the confidence intervals are representative of the model uncertainty. In this work, we highlight key methodological considerations that enable sound ML analysis and describe a workflow that conforms to these principles. First, we emphasize, via computational simulations using synthetic datasets that arise from a two-phase clinical trial design, the need for a causal diagram to identify the set of variables needed to be included in the analysis. Additionally, we underline the implications of these methodological considerations on the ML results for E-R analysis, as well as the need for a set of good practices to avoid potential pitfalls.

## 2. Methods

### 2.1. Synthetic Dataset

**Figure 1:**
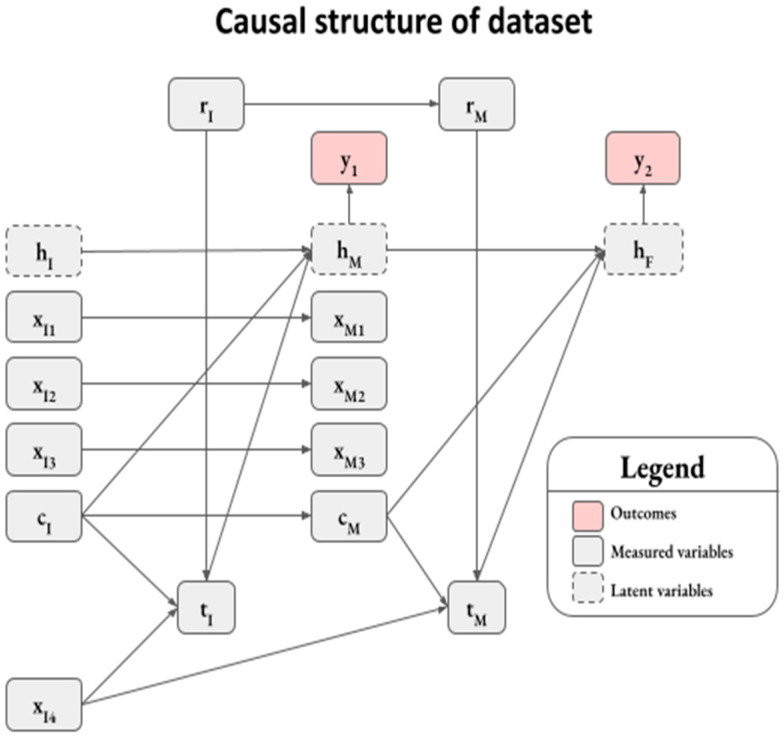
Causal structure of a synthetic clinical trial dataset with two randomized treatment stages (induction (I) and maintenance (M)). The variable subscript *I* refers to variables from the induction stage: **h_I_** represents the health status; **x_I1_**, **x_I2_**, **x_I3_**, and **x_I4_** represent covariates; **r_I_** represents a treatment randomization variable whereby patients were randomized active treatment arm when **r_I_** = 1 or placebo arm when **r_I_** = 0; **c_I_** represents a confounding variable from the beginning of the induction stage; **t_I_** represents drug exposure in the induction stage. The variable subscript *M* refers to variables from the maintenance stage. Correspondingly, **h_M_**, **x_M1_**, **x_M2_**, **x_M3_**, **r_M_**, **c_M_**, and **t_M_** represent the values in the maintenance stage. The variables **y_1_** and **y_2_** represent measured binary outcome variables at the end of the induction and maintenance stages, which are stochastically determined from the latent health status variables **h_M_** and **h_F_**, respectively.

Because this work is methodological in nature, the principles and approaches discussed here are widely applicable to a variety of datasets. In this work, we simply synthesized a dataset where the ground truth functional relationships between variables are known in order to facilitate the illustration of concepts and strengths of a ML-based ER analysis framework. Therefore, we reserved the mathematical details of the synthetic data generation to Appendix A, but here we briefly describe the structure of the dataset (Figure 1).

We synthesized a dataset analogous to a clinical trial with two treatment randomization stages, an induction stage (I) and a maintenance stage (M). This is similar to two-stage trials designs in the IBD therapeutic area [14,15], where there is a need for therapies that provide lasting durable responses. The two-stage trial design allows for assessing the safety and efficacy of a therapy following an initial induction treatment stage as well as a subsequent maintenance treatment stage.

The synthetic dataset had two binary outcome variables, **y_1_** and **y_2_**, which can be interpreted as whether or not patients had favorable outcomes at the end of induction and maintenance, respectively (Figure 1). The outcomes were probabilistically determined from patients’ health status, and terms were included to capture health status at the start of induction (**h_I_**), the start of maintenance (**h_M_**), and the final health status at the end of maintenance (**h_F_**). We considered health status as latent variables that were not directly measurable, and thus not available for ML-based analyses. Treatment randomization for induction and maintenance was determined by the Bernoulli random variables **r_I_** and **r_M_**, respectively, while **t_I_** and **t_M_** reflect drug exposure during the two stages, respectively. Similar to actual clinical trials, only the subset of patients on the active treatment arm during induction (**r_I_** = 1) continued onto the maintenance stage. The dataset includes confounding factors, **c_I_** and **c_M_**, that impact both the exposure and outcome in the induction and maintenance stages, respectively. Other random variables were included in the dataset with a random covariance structure (i.e., **x_I1_**–**x_I4_** and **x_M1_**–**x_M3_**); however, these variables did not have a causal impact on health status or outcomes.

The final synthetic dataset consists of 2000 patients, of whom 40.2% had a favorable outcome at the end of induction (i.e., **y_1_ = 1**). From this subset, 943 patients continued onto the maintenance stage, and 78.2% of patients had a favorable outcome at the end of maintenance (i.e., **y_2_ = 1**) (Figure S1). While we utilized a complex data structure to illustrate the principles of variable selection through the use of causal diagrams and to demonstrate how the proposed ML E-R framework adjusts for confounding effects, we note these principles are applicable as well to simpler trial settings.

### 2.2. Machine Learning

In this analysis, we synthesized data with nonlinear relationships between explanatory variables and outcomes to demonstrate the utility of a ML framework to characterize these relationships. Specifically, we created three models aimed at characterizing the exposure response relationships between t_I_ and y_1_, t_M_ and y_2_, and t_I_ and y_2_ (Figure 1), referred to as the induction model, maintenance-only model, and maintenance from induction model, respectively. The selection of variables for these models are discussed in the Results section (Selection of explanatory variables for ML-based E-R analysis). We utilized XGBoost binary classification algorithms for these analyses, which is a non-parametric tree-based ML algorithm [16]. While we demonstrated the utility of the XGBoost algorithm in relatively simple synthetic datasets in this work, XGBoost has the advantages of scaling well with many explanatory variables, handling missing values, and working well with heterogeneously distributed data.

### 2.3. SHAP Analysis

Shapley Additive Explanations (SHAP) is a ML explainability framework with theoretical underpinnings in cooperative game theory [10]. SHAP analysis decomposes the marginal effect of explanatory variables (SHAP values) on ML predictions. For consistency with the data generation process, we extracted SHAP values in the log odds rather than probability domain using the SHAP package [10]. We denoted terms with a “hat” notation when they related to model-estimated terms. In the SHAP formulation, a model prediction $\hat{f}(x)$ is equal to an estimated expected value (${\hat{\phi }}_{0}$) plus the sum of SHAP values of all explanatory variables, as shown in (1):

(1) $$\hat{f}(x)={\hat{\phi }}_{0}+\sum_{v\in S}^{}{\hat{\phi }}_{v}$$

where *S* represents the set of explanatory variables, and ${\hat{\phi }}_{v}$ is the SHAP value for the explanatory variable *v*.

### 2.4. Ground Truth Marginal Effects

Utilizing synthetic data enabled comparison of model-estimated marginal effects (SHAP values) with known ground truth marginal effects of explanatory variables. A detailed mathematical description of how ground truth marginal effects were calculated can be found in Appendix A. In brief, the synthetic outcomes of the dataset were generated by defining the impact of explanatory variables on outcomes of the log odds domain, which can be thought of as the ground truth marginal effects of the explanatory variables. The ground truth marginal effects were mean subtracted to enable direct comparison with SHAP values (see Appendix A for details).

## 3. Results

In this section, we propose a set of good practices necessary for using ML and SHAP values for E-R analysis and the computational results of adopting them within the ML workflow. In each of the subsections, we describe in detail the computational steps involved and illustrate (via the synthetic data) their importance by comparing to the alternate results if the proposed steps are not followed.

### 3.1. Generating Unbiased Predictions and SHAP Values

**Figure 2:**
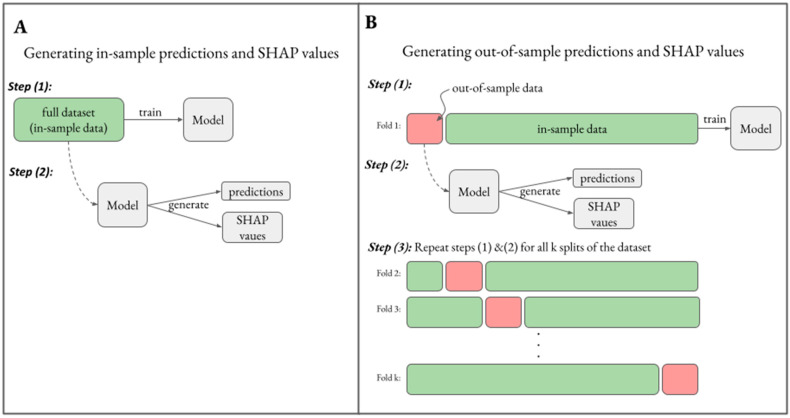
Schematic to generate in- vs. out-of-sample predictions and SHAP values. (**A**) To generate in-sample predictions and SHAP values, the full dataset is used both to train a model and to generate predictions and SHAP values. (**B**) Out-of-sample predictions and SHAP values are generated using a k-fold cross-validation schema, whereby in each fold, a model is trained on in-sample training data and predictions and SHAP values are generated on out-of-sample data.

**Figure 3:**
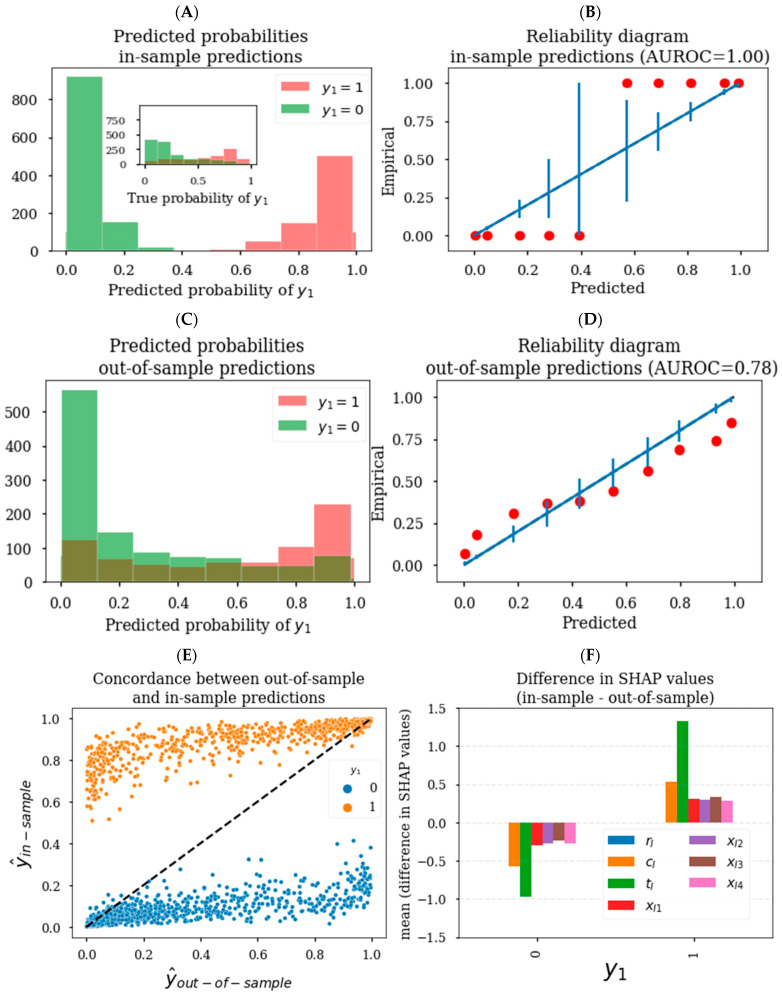
In-sample vs. out-of-sample prediction reliability and differences in SHAP value estimates (without hyperparameter tuning). Distribution of ŷ_in-sample_ (**A**) and ŷ_out-of-sample_ (**C**) colored by **y_1_** status. Inset in (**A**) shows the distribution for the true probability (***σ***(**h_M_**)). (**B**) Reliability of ŷ_in-sample_ and (**D**) ŷ_out-of-sample_ with empirical rates of **y_1_**, with the binning schema being identical to the corresponding ŷ_in-sample_ (**A**) and ŷ_out-of-sample_ (**C**) distribution plots. Vertical bars in (**B**,**D**) represent the expected 95% binomial confidence intervals based on binned ŷ_in-sample_ and ŷ_out-of-sample_ values, respectively. (**E**) Concordance between ŷ_in-sample_ and ŷ_out-of-sample_ demonstrating predominantly higher ŷ_in-sample_ values when **y_1_** = 1, vice versa when **y_1_** = 0. (**F**) Bar plot demonstrating mean in-sample SHAP values were greater **y_1_** = 1, vice versa when **y_1_** = 0 for each of the induction model features.

While ML models have the flexibility to describe complex nonlinear relationships, this property could result in overfitting on training data unless proper care is taken [17]. We first demonstrated how an XGBoost model generates biased predictions on training data. Training data or in-training sample data (henceforth referred to as in-sample data) refers to data that was used to train a model. This contrasts with out-of-sample data which refers to data that was not utilized to train a model. According to the schematic in Figure 2A, we utilized the induction model with explanatory variables {**x_I1_**, **x_I2,_ x_I3_**, **x_I4_**, **c_I_**, **t_I_**} to train an XGBoost model to predict the binary outcome **y_1_**. Figure 3A shows that when we generated model-based predicted probabilities on in-sample data (ŷ_in-sample_), there was a clear separation dependent on **y_1_** status such that predicted probabilities for **y_1_** = 1 were greater than predicted probabilities for **y_1_** = 0 in all cases. This represents a perfect classification of **y_1_**, with classification performance of 1.0 when evaluated using the area under the receiver operator curve (AUROC); however, in this synthetic dataset, the ground truth probabilities are known and cannot be dichotomized exactly by **y_1_** status (see inset in Figure 3A). Using the ground truth **y_1_** probabilities, the maximum theoretical performance was AUROC = 0.84 rather than 1.0, suggesting the model performed implausibly well due to overfitting. Moreover, in Figure 3B, we assessed the reliability of the model, which relates to how well the predicted probability distribution relates to the true probability distribution. Here, the binned ŷ_in-sample_ did not reliably correspond to the empirical rates of **y_1_**. In real datasets, the ground truth probabilities are unknown; however, through this synthetic example we demonstrated that ŷ_in-sample_ can be biased toward the actual outcomes, which can yield inflated performance metrics and unreliable predictions.

A standard approach to generate unbiased predictions is to use a k-fold cross-validation schema, as illustrated in Figure 2B. In this methodology, a $\frac{1}{k}$th of the dataset is split into out-of-sample data that is reserved to generate predictions and inferences, while the remaining $\frac{k-1}{k}$th of the data is in-sample data utilized to train a model. Having the separation between in-sample and out-of-sample data ensures that models do not have the ability to overfit on out-of-sample data upon which predictions are generated. This process is repeated for k-folds to cover the generation of predictions across the entire dataset, which is known as cross-validation. We utilized a 10-fold cross-validation approach to generate out-of-sample predictions (ŷ_out-of-sample_), which are specifically predictions on data that was not used to train the model. In contrast to ŷ_in-sample_, the ŷ_out-of-sample_ distribution was not perfectly separable based on **y_1_** status (Figure 3C). The classification performance based on ŷ_out-of-sample_ was AUROC = 0.78, which was plausibly less than the theoretical maximum of 0.84. ŷ_in-sample_ were generally greater than ŷ_out-of-sample_ when **y_1_** = 1 and vice versa when **y_1_** = 0 (Figure 3E), further illustrating the bias in ŷ_in-sample_ toward the outcomes. Because SHAP analysis is utilized to explain model predictions according to Equation (1), SHAP values corresponding to ŷ_in-sample_ were on average greater than SHAP values corresponding to ŷ_out-of-sample_ when **y_1_** = 1 and vice versa when **y_1_** = 0 (Figure 3F). All together, this highlights that ŷ_in-sample_ can be biased toward the outcomes, and explaining biased predictions using SHAP analysis can perpetuate these biases onto SHAP values. Ensuring a separation between in-sample training data and out-of-sample data for prediction and inference generation through a cross-validation approach can help to mitigate biases in predictions and inferences.

Figure 3D demonstrates that ŷ_out-of-sample_ provided more reliable predictions than ŷ_in-sample_ (Figure 3B), with points closer to the line of parity for ŷ_out-of-sample_. Nevertheless, for many of the binned ŷ_out-of-sample_, the corresponding empirical rates of **y_1_** were not within the expected 95% binomial confidence intervals estimated from the predictions, suggesting that the ŷ_out-of-sample_ still exhibited poor reliability. Next, we demonstrated how hyperparameter tuning improved the reliability of predictions and SHAP-based inferences.

### 3.2. Generating Reliable Predictions and SHAP Values

ML models can be prone to overfit on training data. As such, it is standard practice to keep a strict separation between in-sample data used for model training and out-of-sample data used for assessing model performance and generating inferences. This separation prevents predictions and inferences from being biased toward the outcomes, but it does not preclude overfitting to the training data and consequent poor generalizability. ML models have hyperparameters that can be tuned to optimize the bias–variance tradeoff to improve generalizability. As each ML model has its own set of hyperparameters, it is beyond the scope of this work to discuss how specific hyperparameters alter model training. Moreover, there are many hyperparameter tuning methods with their own strengths and limitations [18]. For this analysis, we utilized an efficient Bayesian hyperparameter search algorithm using the hyperopt package to optimize AUROC using 5-fold cross-validation and 25 search iterations in the XGBoost hyperparameter search space defined in Supplementary Table S1.

**Figure 4:**
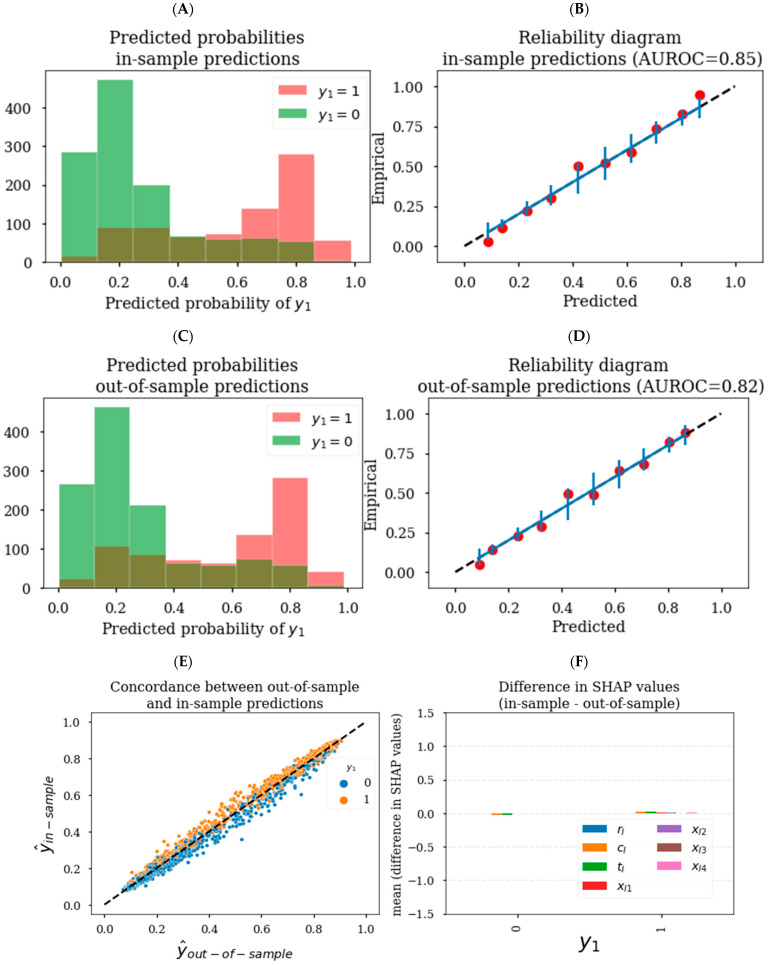
In- vs. out-of-sample prediction reliability and differences in SHAP value estimates (with hyperparameter tuning). Distribution of ŷ_in-sample_ (**A**) and ŷ_out-of-sample_ (**C**) colored by **y_1_** status. Reliability of (**B**) ŷ_in-sample_ and (**D**) ŷ_out-of-sample_ with empirical rates of **y_1_**, with the binning schema being identical to the corresponding (**A**) ŷ_in-sample_ and (**C**) ŷ_out-of-sample_ distribution plots. Vertical bars in (**B**) and (**D**) represent the expected 95% binomial confidence interval based on binned ŷ_in-sample_ and ŷ_out-of-sample_ values, respectively. (**E**) Concordance between ŷ_in-sample_ and ŷ_out-of-sample_ demonstrating slightly higher ŷ_in-sample_ values when **y_1_** = 1, vice versa when **y_1_** = 0 in general. (**F**) Bar plot demonstrating mean in-sample SHAP values were similar for each of the induction model features.

Hyperparameter tuning to optimize cross-validation model performance can improve the generalizability of a model. Figure 4A demonstrates there was not an exact dichotomization of ŷ_in-sample_ based on **y_1_** status after hyperparameter tuning as was seen without hyperparameter tuning (Figure 3A). ŷ_in-sample_ also tended to be much more reliable after hyperparameter tuning (Figure 4B) compared to without hyperparameter tuning (Figure 3B), with empirical rates of **y_1_** closely corresponding to predicted probabilities. In this example, the distribution and reliability of ŷ_in-sample_ was similar to that of the ŷ_out-of-sample_ after hyperparameter tuning (Figure 4C,D). However, even after hyperparameter tuning, there were slight differences in the predictions, whereby ŷ_in-sample_ were generally greater than ŷ_out-of-sample_ when **y_1_** = 1 and vice versa when **y_1_** = 0 (Figure 4E). These differences in ŷ_in-sample_ vs. ŷ_out-of-sample_ led to minor differences in classification performance in terms of AUROC (0.85 vs. 0.82, respectively). Given that model performance based on ŷ_in-sample_ was slightly above the maximum theoretical performance of 0.84 suggests that ŷ_in-sample_ may still be biased toward the outcome due to overfitting. We show in Figure 4F that SHAP values corresponding to ŷ_in-sample_ vs. ŷ_out-of-sample_ were similar for this dataset after hyperparameter tuning. However, in general, there is no guarantee that ŷ_in-sample_ and in-sample SHAP values would be unbiased after hyperparameter tuning for other datasets and other ML models. Therefore, we suggest as a best practice to generate predictions and SHAP-based inferences on out-of-sample data that the model has not been trained on.

### 3.3. Selection of Explanatory Variables for ML-Based E-R Analysis

In this analysis, ML was utilized to estimate the marginal effect of exposure upon response variables of interest. The selection of explanatory variables is an important step towards generating the desired inferences from a ML-based E-R analysis, and a causal diagram such as Figure 1 can guide this step [19,20]. It is important for a model to account for potential confounders, which are variables that impact exposure and response (e.g., **c_I_** and **c_M_**). Failure to account for confounding can lead to biased estimates of an E-R relationship or, in extreme cases, lead to the false conclusion that an E-R relationship exists when there is none. Explanatory variables that can explain the variability in response are typically included in traditional E-R analyses to improve the precision of inferences. ML models can generally accommodate a large number of explanatory variables and multicollinearity, and this can be advantageous when attempting to account for all potential confounding variables. Of note, it is important to use the causal diagrams such as Figure 1 when deciding which variables to leave out of the analysis.

To examine the effect of exposure **t_I_** on outcome **y_1_**, we utilized explanatory variables {**x_I1_**, **x_I2,_ x_I3_**, **x_I4_**, **c_I_**, **t_I_**} in the induction model. To examine the effect of **t_M_** on **y_2_**, we utilized explanatory variables {**x_M1_**, **x_M2,_ x_M3_**, **c_M_**, **t_M_, y_1_**} in the maintenance-only model. One could include baseline variables such as **c_I_** and **t_I_** in the maintenance-only model, which would better estimate the latent variable **h_M_**, and thereby improve predictive performance and precision of inferences. However, in this case, inferences drawn regarding the effects of **c_I_** and **t_I_** on **y_2_** should be interpreted with caution since they would partially be subsumed by **y_1_**. Lastly, to examine the effect of **t_I_** on **y_2_**, we utilized the induction stage explanatory variables {**x_I1_**, **x_I2,_ x_I3_**, **x_I4_**, **c_I_**, **t_I_**} to predict **y_2_** in the maintenance from induction model. Specifically, we intentionally did not include **y_1_** in the maintenance from induction model because including **y_1_** would be expected to partially mask the effect of **t_I_** upon **y_2_**.

### 3.4. SHAP Analysis to Infer Functional Relationships

**Figure 5:**
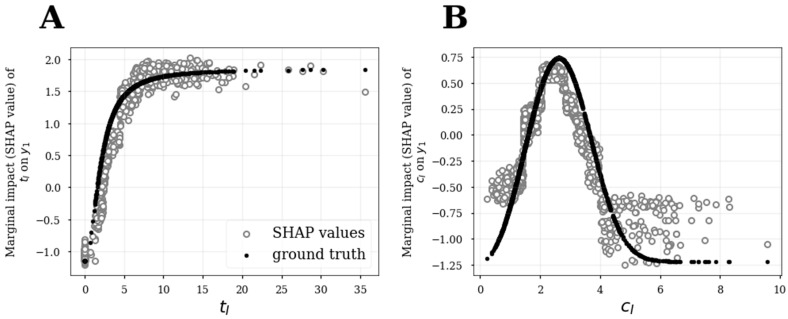
SHAP dependence plots for **t_I_** (**A**) and **c_I_** (**B**) in the induction model. Out-of-sample SHAP values (gray circles) were generated using 10-fold cross-validation and compared to ground truth marginal effects (black dots).

SHAP dependence plots can be utilized to quantitatively characterize how a response depends upon an explanatory variable which lends itself to characterizing E-R relationships. In this analysis, SHAP values were generated in the log odds domain on out-of-sample data using 10-fold cross-validation after hyperparameter tuning to generate unbiased and reliable SHAP values. In each of the SHAP dependence plots, the SHAP value for a given explanatory variable *v* (which represents the estimated marginal effect of the *v* on response) was plotted against the value of *v*. Figure 5 depicts the key dependence plots for the induction model. The estimated E-R relationship on the induction model (Figure 5A) closely approximated the ground truth marginal effects of **t_I_** on **y_1_**. SHAP analysis on the ML model also captured the inverse U-shaped confounding effect of **c_I_** on **y_1_** (Figure 5B), highlighting the ability of non-parametric ML models to capture potential nonlinear relationships; however, poor approximations to the ground truth were apparent at the tail ends of the **c_I_** distribution. While these SHAP dependence plots reveal the functional relationships between explanatory variables and response, it is challenging to assess wherein and to what extent uncertainties in the functional relationships exist. To address this concern, we next discuss how bootstrap analysis can be utilized to estimate confidence intervals around SHAP values.

### 3.5. Realistic Estimation of Confidence Intervals

Quantification of uncertainty in SHAP values can help one address important questions in the learned functional relationships, including whether there may be biases in the model inferences and whether estimated functional relationships are statistically significant. We utilized a nonparametric bootstrap-based approach to estimate confidence intervals for SHAP values. In this approach, we draw *N* samples from our datasets with replacement to train an XGBoost model to predict a response, where *N* is the number of patients in the dataset. SHAP values are then estimated on the out-of-sample data. This process is repeated for an arbitrarily large number of bootstrap iterations (500 iterations in this analysis), from which we estimated the 95% confidence intervals for SHAP values.

Sampling with replacement preserves sampling independence, is necessary for the variability of in-sample datasets if drawing *N* samples and is a standard approach that can provide realistic confidence interval estimates [21]. In contrast, subsampling via sampling without replacement requires selecting an arbitrary training vs. test split size which can lead to unrealistically small or unnecessarily large confidence intervals and/or poor estimations of functional relationships if the training size is too small or too large. Confidence intervals may not necessarily capture the mean ground truth marginal effects due to model and/or data limitations.

**Figure 6:**
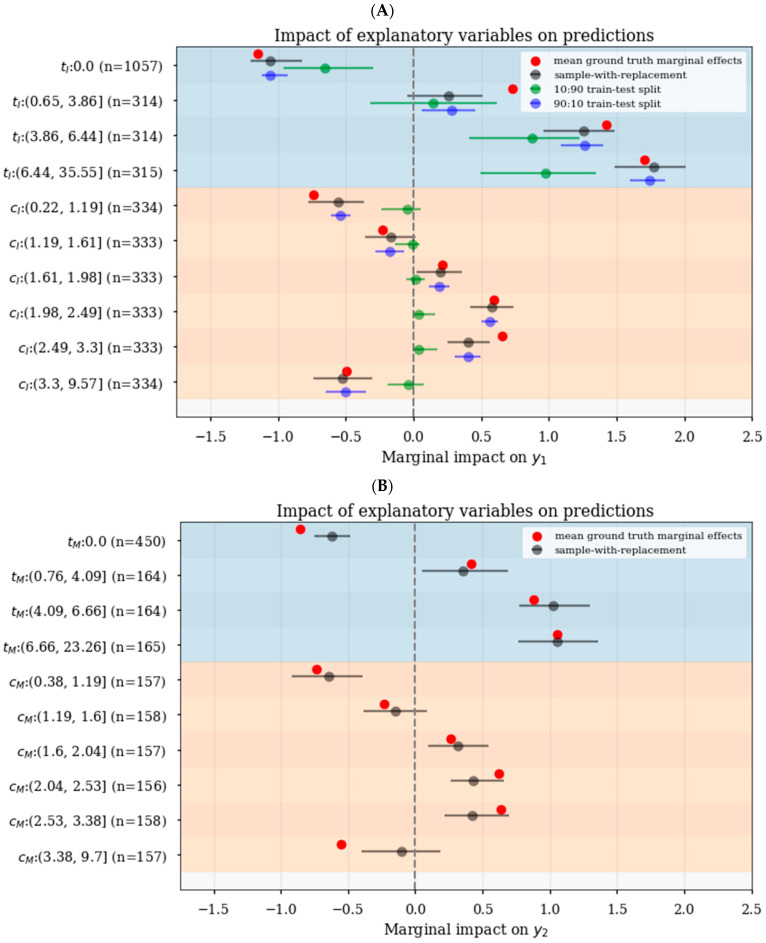
Realistic estimation of confidence intervals for SHAP values with bootstrap sample with replacement strategy. (**A**) Compared to sample with replacement approach (black), the 10:90 train-test split method (green) yielded larger confidence intervals for SHAP values or poor estimation of functional relationships, while the 90:10 train–test–split method (blue) yielded unrealistically small confidence intervals in the induction model. The confidence intervals using the sampling with replacement approach did not perfectly capture all the ground truth marginal effects (red) in the induction (**A**) nor the maintenance-only models (**B**).

The forest plot in Figure 6A demonstrates that the 95% confidence intervals generated using various approaches. In particular, the proposed bootstrapped sampling with replacement approach for **t_I_** and **c_I_** in the induction model is shown in black; in comparison, the 10:90 train–test–split sampling without replacement approach (shown in green) poorly approximated the ground truth effects of **t_I_** and **c_I_** and yielded much larger confidence intervals for **t_I_**; finally, the 90:10 train–test–split sampling without replacement approach (shown in blue) yielded much smaller and unrealistic confidence intervals for both **t_I_** and **c_I_** compared to sampling with replacement (black). While the ML model was imperfect and, hence, confidence intervals using the sampling with replacement approach did not perfectly capture ground truth marginal effects in the induction nor the maintenance-only models (Figure 6A,B, respectively), this computational experiment demonstrated the need for the correct sampling approach to ensure good estimation of the uncertainties in the inferred relationships.

### 3.6. Bootstrapped Feature Dependence Plots

**Figure 7:**
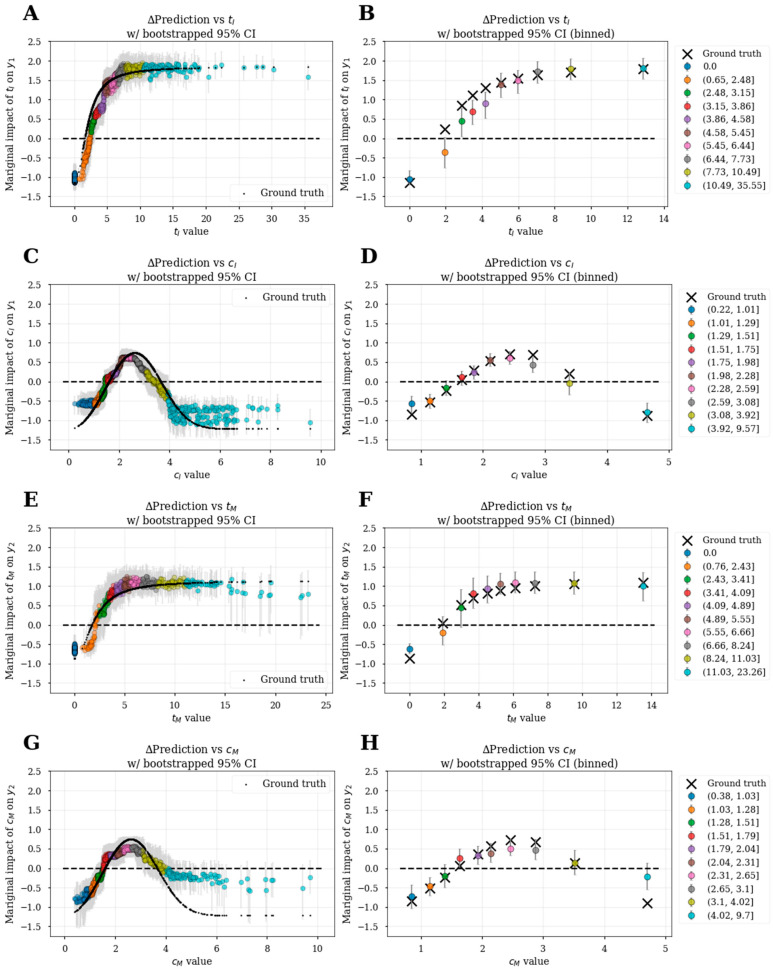
Bootstrapped feature dependence plots for explanatory variables in the induction (**A**–**D**) and maintenance-only (**E**–**H**) models. Left panels show individual-level bootstrapped feature dependence plots (**A**,**C**,**E**,**G**) colored by deciles of explanatory variable values compared to ground truth marginal effects (black dots). Binned data is summarized in corresponding right panels (**B**,**D**,**F**,**H**), which shows the mean SHAP values against the median explanatory variable values and the mean ground truth marginal effects (X). Gray error bars represent 95% confidence intervals.

**Figure 8:**
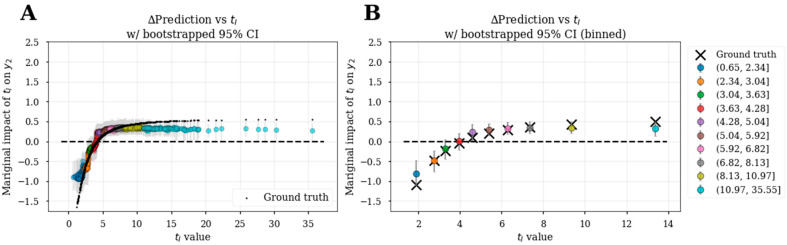
Bootstrapped feature dependence plots for the effect of **t_I_** on **y_2_** from the maintenance from induction model. (**A**) Individual-level bootstrapped feature dependence plots colored by deciles of **t_I_** values compared to ground truth marginal effects (black dots). Binned data from (**A**) is summarized in (**B**), which shows the mean ${\hat{\phi }}_{tI}$ against the median **t_I_** values and the mean ground truth marginal effects (X). Gray error bars represent 95% confidence intervals.

Using the bootstrap (sampling with replacement) approach, we estimated the mean and 95% confidence intervals for all SHAP values. This was utilized to generate bootstrapped feature dependence plots in Figure 7 and Figure 8. Like Figure 5, bootstrapped feature dependence plots can be utilized to characterize the functional relationships between explanatory variables and responses. Additionally, they allow for uncertainty characterization within the functional relationships. In the left panels, the mean and 95% confidence intervals SHAP values of individual patients are plotted against the explanatory variable value. The colors represent different decile bins of the explanatory variables, and the right panels summarize the binned data with the mean and 95% confidence intervals SHAP values of each bin versus median explanatory variable values.

While the bootstrapped feature dependence plots for the induction model are shown in Figure 7A,D, Figure 7A,B demonstrate that the estimated effects of **t_I_** on **y_1_** were comparable to the ground truth and the model misestimated effects for the 2nd–4th quantile groups, with ground truth marginal effects outside of the 95% confidence intervals (Figure 7B). The effect of **c_I_** on **y_1_** was well captured in Figure 7C,D with minor deviations outside of the 95% confidence intervals (Figure 7D). The bootstrapped feature dependence plots for the maintenance-only model are shown in Figure 7E–H. The effect of **t_M_** on **y_2_** was well captured (Figure 7E,F) with the ground truth marginal effects within the 95% confidence interval for all bins except the placebo group (**t_M_** = 0) (Figure 7F). The effect of **c_M_** on **y_2_** was well-captured (Figure 7G,H) with the ground truth marginal effects within the 95% confidence interval for all except the last bin (Figure 7H).

These bootstrapped feature dependence plots on simulated data demonstrate that the XGBoost model can capture nonlinear E-R and confounding relationships. The fidelity of estimations to ground truth is expected to be dataset dependent, but also dependent upon the explanatory variables included in the model. In the maintenance from induction model, we were interested in inferring the effect of **t_I_** on **y_2_**. According to the causal diagram (Figure 1), there is no direct relationship between **t_I_** and **y_2_** but the effect of **t_I_** is mediated through the latent variable **h_M_**. To estimate the effect of **t_I_** on **y_2_**, we did not include **y_1_** as an explanatory variable to predict **y_2_** in the maintenance from induction model as previously mentioned (see the section Selection of explanatory variables for ML-based E-R analysis). Using this approach, we show that the inferred **t_I_**-**y_2_** functional relationship closely matched the ground truth (Figure 8), with slight deviations at the extremes of the **t_I_** distribution that lay outside the 95% confidence intervals.

## 4. Discussion

ML models are inherently non-parametric and can work well in predicting targets when nonlinear relationships exist between explanatory variables and outcomes such as an Emax or an inverted U-shaped E-R relationship. Rather than relying on the expertise of a modeler to define the functional form of relationships, using ML the functional relationship can be derived in a data-driven approach. However, in contrast to well-established statistical methodologies for the analysis of E-R relationships, the use of ML models and SHAP values for E-R analysis [9] is nascent; hence, there is a need for understanding the potential pitfalls of such ML-based approaches, and consequently establish a set of good practices aimed at overcoming them.

In this work, we highlight the importance of the following components in the ML workflow in order to ensure accurate, unbiased results: (1) perform SHAP analysis only on out-of-sample data; (2) perform hyperparameter tuning and check model reliability; (3) generate realistic confidence intervals via appropriate sampling with replacement; (4) leverage causal diagrams to determine which variables should be incorporated into the ML model. In particular, we utilized synthetic binary classification datasets that mimic a two-phase clinical trial, with known functional relationships between explanatory variables and outcomes and demonstrated the results using the tree-based XGBoost ML models for binary classification in conjunction with the SHAP explainability framework. For each of the above mentioned components of the ML workflow, we showed via synthetic data the perils if the proposed good practices are not followed, including: (1) over- and under-estimation of the E-R effects; (2) over- and under-estimation of the predicted confidence intervals; and (3) use of inappropriate variables that either leave out important confounders or mask the true E-R relationship. Finally, we showed that even with a challenging synthetic dataset that mimics a two-phase clinical trial which exhibits nonlinear and/or non-monotonic E-R and confounding relationships, the ML model was nevertheless able to adequately infer the underlying relationships. This result suggests that the proposed ML workflow is adequate for the E-R task at hand and provides a promising alternative to parametric statistical modeling, which would be challenging to perform due to the nonlinearity and non-monotonicity involved.

The application of these proposed practices should enable sound model-based inferences. It is important to note that while ML models have favorable properties that may accurately estimate E-R relationships even in the setting of strong confounding effects, ML-based inferences are subject to certain biases and limitations, as with any other model [7]. While we have addressed key methodological factors that may impact the results of E-R analysis, there are areas for further development that are beyond the scope of the current work. In particular, the confluence of causal graphs and SHAP analysis offers a way to advance these concepts [22] and remains a topic for future research.

## Data Availability

Data sharing is not applicable to this article.

## Supplementary Materials

The following supporting information can be downloaded at: https://www.mdpi.com/article/10.3390/pharmaceutics15051381/s1, Figure S1: Characteristics of synthetic datasets; Table S1: Hyperparameter search space for XGBoost models.

## Appendix A

### Appendix A.1. Data Generation Process

Synthetic patient data was generated by sampling variables from a multivariate normal distribution, followed by data transformation, and, finally, synthesizing outcomes. We use capital letters *X* = (*H_I_*, *X*_*I*1_, *X*_*I*2_, *X*_*I*3_, *X*_*I*4_, *C_I_*, *C_M_*, *T_I_*, *T_M_*) to denote random variables, while lower case letters (e.g., **h_I_**, **x_I1_**, **x_I2_**, … **r_M_**, **c_M_**, and **t_M_**) denote the associated realized values (after sampling and/or transformations have taken place). For each patient, a nine-dimensional vector was sampled from a multivariate normal distribution with a vector of mean values μ and the covariance matrix ∑ defined in (A1). The covariance structure was selected to explicitly impose correlation between the confounding variable **c_I_** and **t_I_** as well as **c_M_** and **t_M_**. The covariance structure also imposed correlations between the health status **h_I_** and the set of covariates {**x_I1_**, **x_I2_**, **x_I3_**}, as well as between the covariate **x_I4_** and exposure variables {**t_I_**, **t_M_**}.

(A1) $$X∼N_{9}\left(\mu ,\Sigma \right)\;\left[\begin{array}{c} H_{I}\\ X_{I1}\\ X_{I2}\\ X_{I3}\\ X_{I4}\\ C_{I}\\ C_{M}\\ T_{I}\\ T_{M} \end{array}\right]∼N\left(\left[\begin{array}{c} -2\\ ln\left(5\right)\\ ln\left(5\right)\\ ln\left(5\right)\\ ln\left(5\right)\\ ln\left(2\right)\\ ln\left(2\right)\\ ln\left(5\right)\\ ln\left(5\right) \end{array}\right],\left[\begin{array}{ccccccccc} 0.0 & -0.1 & 0.1 & -0.1 & 0.0 & 0.1 & 0.0 & 0.0 & 0.0\\ -0.1 & 0.2 & 0.0 & 0.0 & 0.0 & 0.0 & 0.0 & 0.0 & 0.0\\ 0.1 & 0.0 & 0.2 & 0.0 & 0.0 & 0.0 & 0.0 & 0.0 & 0.0\\ -0.1 & 0.0 & 0.0 & 0.2 & 0.0 & 0.0 & 0.0 & 0.0 & 0.0\\ 0.0 & 0.0 & 0.0 & 0.0 & 0.1 & 0.0 & 0.0 & 0.3 & 0.3\\ 0.1 & 0.0 & 0.0 & 0.0 & 0.0 & 0.1 & 0.2 & 0.2 & 0.0\\ 0.0 & 0.0 & 0.0 & 0.0 & 0.0 & 0.2 & 0.1 & 0.0 & 0.2\\ 0.0 & 0.0 & 0.0 & 0.0 & 0.3 & 0.2 & 0.0 & 0.1 & 0.0\\ 0.0 & 0.0 & 0.0 & 0.0 & 0.3 & 0.0 & 0.2 & 0.0 & 0.1 \end{array}\right]\right)$$

### Appendix A.2. Induction Stage

The formulae defining the independent variables in the induction stage are shown in (A2). Initial health state (**h_I_**) was defined as the realized value of *H_I_*. The random variables *X*_*I*1_, *X*_*I*2_, *X*_*I*3_, *X*_*I*4_, and *C_I_*, were log transformed to generate **x_I1_**, **x_I2_**, **x_I3_**, **x_I4_**, and **c_I_**, respectively. The treatment randomization term **r_I_** was defined as a Bernoulli random variable with 0.5 probability. Drug exposure in the induction stage (**t_I_**) was defined as ln(*T_I_*) or 0, which was conditional upon **r_I_**. While log transformation was not strictly necessary, it aligns with the non-negative distribution of variables that are common for biomarkers and drug exposure in clinical datasets.

Definition of independent variables in the induction stage:

(A2) $$\begin{array}{l} h_{I}\;≔H_{I}\\ x_{I1}≔X_{I1}\\ x_{I2}≔ln\left(X_{I2}\right)\\ x_{I3}≔ln\left(X_{I3}\right)\\ x_{I4}≔ln\left(X_{I4}\right)\\ c_{I}\;≔ln\left(C_{I}\right)\\ r_{I}\;≔Bernoulli\left(0.5\right)\\ t_{I}≔\left\{\begin{array}{l} ln\left(T_{I}\right),\;\;r_{I}=1\\ 0,\;\;otherwise \end{array}\right. \end{array}$$

The measured dependent variable for the induction stage was a binary variable **y_1_** that indicated whether a patient had a favorable outcome at the end of the induction stage. The value **y_1_** was stochastically determined from the unmeasured latent variable **h_M_**, which represents the health status at the end of the induction stage and can be simultaneously interpreted as the log odds of a favorable outcome. The sigmoid function (A3) was used to convert **h_M_** to the probability of a favorable outcome:

(A3) $$\sigma \left(x\right)=1/\left(1+e^{-x}\right)$$

We simulated **c_I_** to have a nonlinear effect on **h_M_**; the specific functional form is unimportant, but we parameterized the **c_I_** effect on **h_M_** using a Weibull probability density function (A4).

(A4) $$W\left(x\right)=10{\left(\frac{x}{3}\right)}^{2}e^{-{\left(x/3\right)}^{3}}$$

We implemented a saturable effect of drug exposure **t_I_** on **h_M_** using the Hill function (A5) with k_m_ = 2 and Hill coefficient = 2. In the induction stage, we set *E_max_* = 3.

(A5) $$Hill\left(x\right)=\frac{E_{max}}{1+{\left(2/x\right)}^{2}}$$

The nonlinear impacts of **c_I_** and **t_I_** on **h_M_** were added to **h_I_,** along with a constant deterioration of health (−1) according to Equation (A6a). Lastly, **y_1_** was generated as a Bernoulli random variable with ***σ***(**h_M_**) probability given in (A6b).

Definition of dependent variables in the induction stage:

(A6a) $$h_{M}\;≔h_{I}+W\left(c_{I}\right)+Hill\left(t_{I}|E_{max}=3\right)-1$$

(A6b) $$y_{1}\;≔Bernoulli\left(\sigma \left(h_{M}\right)\right)$$

### Appendix A.3. Maintenance Stage

The dataset for the maintenance stage was synthesized only for the subset of patients in the active treatment arm of the induction stage (i.e., **r_I_** = 1), which was 943 out of 2000 synthetic patients. The formulae defining the independent variables in the maintenance stage are shown in (A7). Health status at the start of maintenance, **h_M_**, was set to be the health status at the end of induction. The values of variables **x_M1_**, **x_M2,_** and **x_M3_** correspond to the induction stage variables **x_I1_**, **x_I2_**_,_ and **x_I3_**, respectively, with the addition of Gaussian noise (with mean of 0 and standard deviation of 0.01). The values of variables **c_M_**, **r_M_**, and **t_M_** were defined similarly to **c_I_**, **r_I_**, and **t_I_**, respectively, in the induction stage.

Definition of independent variables in the maintenance stage:

(A7) $$\begin{array}{l} h_{M}\;≔h_{M}\\ x_{M1}≔x_{I1}+N\left(0,0.01\right)\\ x_{M2}≔x_{I2}+N\left(0,0.01\right)\\ x_{M3}≔x_{I3}+N\left(0,0.01\right)\\ c_{M}\;≔ln\left(C_{M}\right)\\ r_{M}\;≔Bernoulli\left(0.5\right)\\ t_{M}≔\left\{\begin{array}{l} ln\left(T_{M}\right),\;\;r_{M}=1\\ 0,\;\;otherwise \end{array}\right. \end{array}$$

The formulae defining the dependent variables in the maintenance stage are shown in (A8). Dependent variables in the maintenance stage were simulated from the corresponding maintenance variables in a similar manner to the induction stage (A6), except with *E_max_* = 2.

Definition of dependent variables in the maintenance stage:

(A8a) $$h_{F}\;≔h_{M}+W\left(c_{M}\right)+Hill\left(t_{M}|E_{max}=2\right)-1$$

(A8b) $$y_{2}\;≔Bernoulli\left(\sigma \left(h_{F}\right)\right)$$

### Appendix A.4. Ground Truth Marginal Effects of Explanatory Variables

Causal diagrams [19] such as Figure 1 provide a way to graphically represent the dependency relationships of variables and outcomes from which one can determine what variables to include or exclude in the analysis based on the exposure–response relationship that is sought after. Based on Figure 1, we constructed 3 ML models: (1) an induction model that utilized explanatory variables from the beginning of induction (x_I1_, x_I2,_ x_I3_, x_I4_, and c_I_) and exposure at induction (t_I_) to predict y_1_; (2) a maintenance-only model that utilized explanatory variables from the beginning of maintenance (x_M1_, x_M2,_ x_M3_, and c_M_) and exposure at maintenance (t_M_) to predict y_2_; and (3) a maintenance-from-induction model that utilized explanatory variables from the beginning of induction (x_I1_, x_I2,_ x_I3_, x_I4_, and c_I_) and exposure at induction (t_I_) to predict y_2_.

In the induction and maintenance-only models, the marginal effects of explanatory variables were represented by terms in Equations (A6a) and (A8a), respectively. For example, in the induction model, **c_I_** affects the log odds of a favorable outcome according to Weibull function in Equation (A4), while **t_I_** affects the log odds of a favorable outcome according to Hill function in Equation (A5). In the maintenance from induction model, explanatory variables affect **y_2_** through their effects on **h_M_**. Therefore, the terms in (A6a) also represent the marginal effects of **t_I_** and **c_I_** on **y_2_**. Because we compared ground truth marginal effects with model-derived SHAP values, which represent the estimated marginal effects of explanatory variables relative to an expected value, it was necessary to center the ground truth marginal effects of explanatory variables around an expected value. The expected value of the marginal effect of a variable *v* is simply the mean effect of *v* across all N patients in the dataset. We represented the marginal effects with ∆ and *ϕ* to represent marginal effects relative to an expected value (henceforth referred to as ground truth marginal effects) (A9).

(A9) $$\phi_{v_{i}}=\Delta_{v_{i}}-\frac{\sum_{j=1}^{N}\Delta_{v_{j}}}{N}$$

where *ϕ*_vi_ is the ground truth marginal effect of variable *v* on the *i*-th patient, and *N* is the number of patients in the dataset.

For all 3 models, we focused on the ground truth marginal effects on outcomes defined in Equations (A6a) and (A8a), which were the effects of: **t_I_**, **c_I_** on **y_1_**; **c_M_**, **t_M_** on **y_2_**; and **t_I_**, **c_I_** on **y_2_**. Nevertheless, other variables such as **x_I1_**, **x_I2_**, and **x_I3_** have minor utility in predicting outcomes due to correlations with the baseline latent health status variable **h_I_**; we did not compare nor calculate the ground truth marginal effects for these variables in this analysis.
