# Supplementary material for: Machine Learning for Exposure-Response Analysis: Methodological Considerations and Confirmation of Their Importance via Computational Experimentations
Source: Pharmaceutics. 2023 Apr 30;15(5):1381. doi: 10.3390/pharmaceutics15051381 (PMC10221670; doi:10.3390/pharmaceutics15051381)
Supplement: Supplementary file 1 [file pharmaceutics-15-01381-s001.zip › pharmaceutics-2259671-supplementary.pdf]

pharmaceutics

MDPI

Supplementary Materials: Machine Learning for Exposure-Response Analysis: Methodological Considerations and Confirmation of Their Importance via Computational Experimentations

Rashed Harun, Eric Yang, Nastya Kassir, Wenhui Zhang and James Lu *

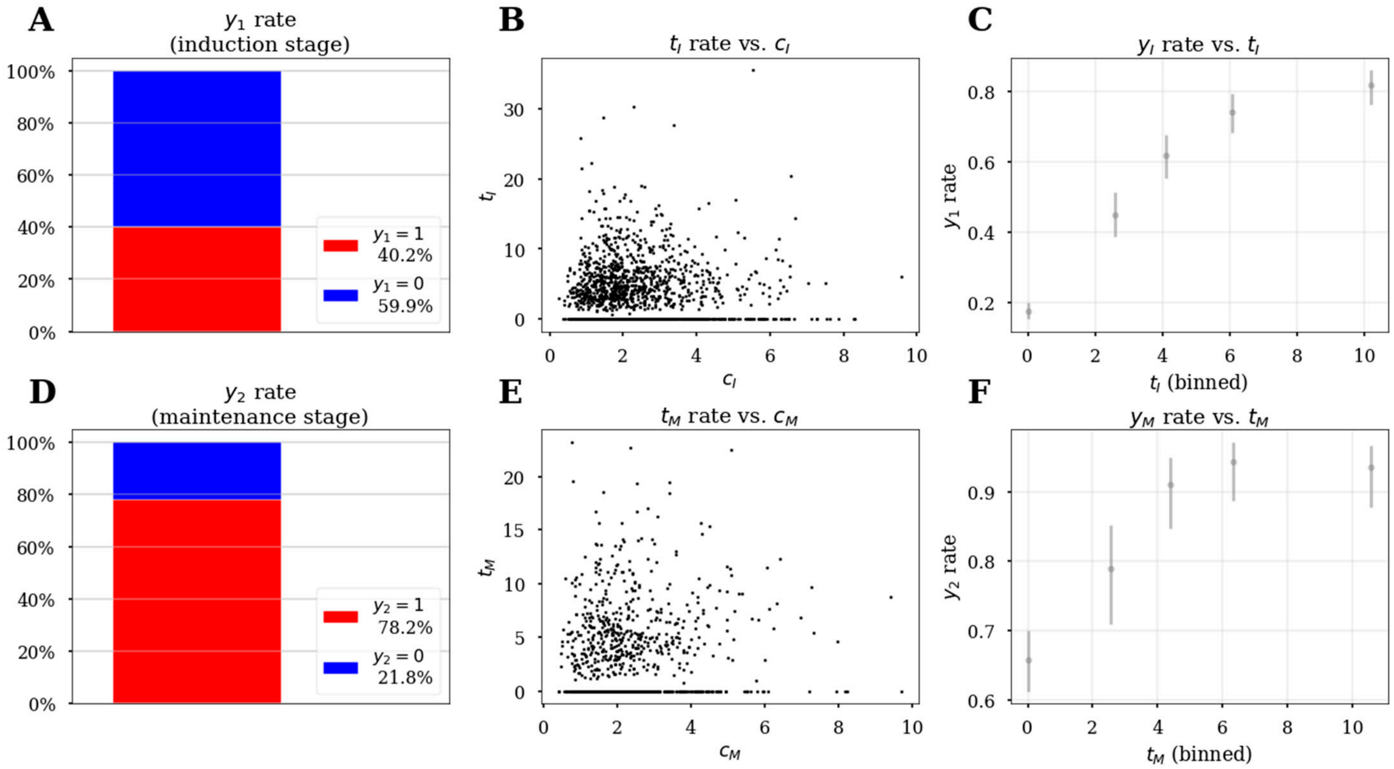

Figure S1. Characteristics of synthetic datasets. Outcome proportions in the induction (**A**) and maintenance (**D**) stages. The relationship between exposure and the confounding variable in the induction (**B**) and maintenance (**E**) stages. The unadjusted exposure response relationship in the induction (**C**) and maintenance (**F**) stages with 95% confidence intervals.

XGboost hyperparameter	Min	max
eta	0.01	.5
max_depth	2	5
min_child_weight	0.01	10
reg_alpha	0.2	10
reg_lambda	0.001	100
subsample	0.6	1

Table S1. Hyperparameter search space for XGBoost models. num_boost_round was set to 100 for all models in this report.
